# Mikomeseng: Leprosy, Legitimacy and Francoist Repression in Spanish Guinea

**DOI:** 10.1093/shm/hkx094

**Published:** 2017-11-03

**Authors:** David Brydan

**Keywords:** leprosy, Francoism, repression, protest, Spanish Guinea

## Abstract

The Mikomeseng leprosy settlement in Spanish Guinea (present-day Equatorial Guinea) was widely promoted during the 1940s and 1950s as the embodiment of the Francoist ‘civilizing mission’ in Africa. Its prominence reflected the important role which colonial health and social policy played in establishing the legitimacy of the Franco regime, and particularly in helping to overcome its international isolation in the immediate post-war era. But a major protest by leprosy sufferers in 1946 revealed the everyday violence underpinning life in Mikomeseng, showing how the language of welfare and social justice which pervaded Francoist propaganda masked the reality of a coercive colonial system. The image of Mikomeseng as the embodiment of benevolent colonial rule was constructed by Francoist experts and officials around a brutally repressive institution, one which encapsulated the violence of Spanish colonial rule in West Africa and of the Franco regime as a whole.

On 18 February 1946 the residents of the Mikomeseng leprosy settlement in Spanish Guinea (present-day Equatorial Guinea) rose up in protest against the brutal conditions they were forced to live under. Mikomeseng, located close to the border of Cameroon, had been established in 1945 as the primary settlement for the estimated 4,000 leprosy sufferers in the colony, part of a new anti-leprosy programme launched by the colonial service of Spain’s Franco regime.^1^ The protest was quickly suppressed by colonial authorities, and its ringleaders were severely punished. But the subsequent investigation revealed the brutality of everyday life in the settlement. As the colonial regime’s own testimonies and reports showed, leprosy sufferers from across Spanish Guinea had been transported to Mikomeseng against their will under brutal conditions, often resulting in death. They had experienced violence, hunger and neglect in the settlement itself, and many had been forcibly separated from their new-born children.

Despite this repressive reality, however, Mikomeseng was widely promoted during the late 1940s and 1950s as the embodiment of the Francoist ‘civilizing mission’ in Africa. A documentary film produced in the same year as the protest and commissioned by Spain’s colonial service presented it as a model institution, providing medical and spiritual care, education and security for its residents, while protecting both new-born children and the wider population from the dangers of infection.^2^ Numerous other articles, books and films promoted Spain’s successful treatment of leprosy in the settlement, aimed both at domestic and international audiences. For the Spanish judge and colonial administrator Rafael Galbe Pueyo, Mikomeseng demonstrated ‘the unprecedented generosity of a colonizing nation’, working quietly and modestly to secure the healthy and prosperous future of its colonial subjects.^3^

Recent research has shown how, despite the hunger, hardship and repression which characterised the first decades of the Franco regime, social policy and the language of social justice were central to Francoist attempts to secure legitimacy and gain popular support.^4^ This paper uses the history of Mikomeseng to argue that colonial health and social policy also played an important role in efforts to establish the legitimacy of the Franco regime during the 1940s and 1950s. These efforts were not solely aimed at a domestic audience. Particularly during the period of international isolation which followed the end of the Second World War, Spanish officials and experts adopted the language of humanitarianism and development deployed by the British and French empires to position Franco’s Spain as a respectable and progressive colonial power.^5^ Mikomeseng, leprosy control and colonial health policy more generally, thus played an important part in efforts to overcome Spain’s post-war isolation.^6^ The prominent role of Francoist experts in international leprosy organisations reflected the wider success of Franco’s Spain in exploiting the idea of its ‘civilizing mission’ in Africa to establish international legitimacy.

Spain’s twentieth-century African colonies were extremely small, limited to Spanish Guinea, a strip of territory along the northern Moroccan coast, and a sparsely inhabited region in the western Sahara.^7^ Nonetheless, Spain’s African empire stood at the heart of Francoist ideology and foreign policy during the early years of the regime. The language of empire had been central to the discourse of Spanish nationalist and right-wing movements since the ‘Disaster’ of 1898, when Spain had lost its remaining Latin American and Asian colonies.^8^ Spain had laid claim to territories in equatorial Africa since the late 1700s, but did not establish full control over Spanish Guinea—the island of Fernando Po (present-day Bioko) and the small mainland region of Rio Muni—until the early twentieth century. Control of the Protectorate of Morocco plunged Spain into a series of brutal and costly colonial conflicts between 1909 and 1927, which helped to form a generation of *africanista* officers and soldiers, including Franco himself, for whom the brutal and brutalising experiences of Morocco shaped their political outlook.^9^

The role of Morocco and Moroccan troops in securing the rebel victory during the Spanish Civil War helped to embed this *africanista* culture at the heart of the Francoist state, and the new regime initially hoped that the Second World War would provide an opportunity to expand Spanish territories in the continent.^10^ These dreams of imperial expansion were quickly dashed, but Africa and Spain’s imperial status continued to permeate Francoist language and thought, particularly in the period before the regime was forced to cede Moroccan independence in 1956. Indeed, the idea of Spain as a progressive imperial power remained an important part of the self-image and diplomatic strategies of the Franco regime right up until the independence of Spanish Guinea in 1968, and of the Spanish Sahara in 1975.^11^

The treatment of leprosy was the ideal vehicle to promote an image of benevolent Francoist imperialism, both due to the disease’s religious symbolism, and to the new developments in leprosy treatment which emerged in the aftermath of the Second World War. Although Gerhard Hansen’s research of the 1870s linking leprosy to the *M. leprae* bacillus had transformed traditional understandings of the disease, its historical association with Christianity, the Catholic Church, the Order of St Lazarus and missionary work meant that religious ideas and practices continued to influence approaches to the disease well into the twentieth century. This was particularly true in Franco’s Spain, where the state was underpinned by a highly conservative ‘National Catholic’ ideology, and where the Church played a central role in national life.^12^ The significance of the disease under the Franco regime was reflected in the new leprosy institutions and regulations which were introduced in Spain between 1943 and 1946, which returned leprosy settlements to the religious orders which had controlled them prior to the Second Republic.^13^

But it was also discernible in the renewed attention given to the disease in Spanish Guinea, where the treatment of leprosy was also dominated by religious orders and religious symbolism. The importance of religion to the Francoist leprosy programme reflected the wider dominance which Christian organisations, beliefs and symbolism continued to exert over the treatment of the disease across colonial Africa. Recent research has shown how missionary groups continued to dominate African leprosy care until well into the 1960s, and how religious organisations took the lead in international anti-leprosy campaigns.^14^ The segregation policies and settlements which characterised much leprosy treatment until after the Second World War provided missionaries and religious orders with the perfect opportunity to evangelise a captive and vulnerable patient population.^15^ Within these institutions the physical deformities of sufferers were taken to symbolise moral and spiritual failings, with care focusing as much on patients’ spiritual as their physical well-being. Megan Vaughan has argued that, although not ‘total institutions’ in the Foucauldian sense, leprosy colonies served to project Christian disease symbolism onto African sufferers, creating a decontextualised ‘leper identity’ which rendered individuals as courageous and stoic bearers of their biblical scourge.^16^

The establishment of the new Mikomeseng settlement in 1945, however, also coincided with one of the key developments in the modern treatment of leprosy. Prior to the Second World War most anti-leprosy programmes had continued to revolve around traditional ideas of settlement and isolation, and medical intervention was limited to the largely ineffective use of chaulmoogra oil. Research carried out in the USA during the Second World War led to the discovery of sulfones, which revolutionised the field by providing the first truly effective treatment against leprosy. The use of sulfones spread across the world in the immediate post-war years and quickly supplanted chaulmooga oil as the principle method of treatment.^17^

Eventually these developments would prompt a shift away from settlements and segregation in the treatment of leprosy, but during the 1950s the success of sulfone treatments within Mikomeseng could be held up by Spanish colonial experts and officials as evidence of the medical and scientific achievements of Francoist rule. The nature of the disease and the visible impact of its cure meant that these ‘achievements’ were written on the healed skin of leprosy sufferers, and could be presented as stark visual evidence of African progress and development under Spanish tutelage.^18^ As Michael Worboys has argued, leprosy treatment served to demonstrate the Christianising mission and the developmental mandate of European colonialism during the twentieth century.^19^ At a time when international critics of the Franco regime denounced it as a backwards and regressive remnant of interwar fascism, Mikomeseng could be used to align Spain with this broader European project by portraying it as a Christian, socially-just, and scientifically-advanced colonial power dedicated to African development.

But the evidence of everyday violence underpinning life in Mikomeseng which emerged after the 1946 protests demonstrates that the language of welfare and social justice which pervaded Francoist propaganda masked the reality of a colonial system built on coercion and repression. Under Francoist rule in Spanish Guinea, a repressive military regime, backed up by the Church, exercised violent and coercive control over the lives of the majority of its African subjects, prioritising the supply of cheap, pliant labour to the major timber and cacao plantations, while strictly limiting basic civic rights and neglecting social services.^20^ This was particularly evident in health policy, where authorities in Spanish Guinea extended a pre-existing regime of sanitary surveillance involving restrictive health passports, strict limits on movement and travel, mandatory medical examination and reporting systems and racially-based intelligence testing.^21^

In these respects, it differed little from the Spanish colonial regimes which had preceded it. But it also mirrored some of the unique characteristics of Francoist repression on the Spanish mainland. Efforts to control the lives of Africans and to mould them into pliant Catholics citizens of the Spanish ‘New State’ bore parallels to the treatment of former Republicans and working-class communities in Spain, often themselves depicted as racially-inferior outsiders. The treatment of leprosy sufferers in Mikomeseng, in particular, echoed the Francoist use of coercive health programmes, the institutionalisation of children from Republican families, and the mass incarceration of political opponents in Spain itself.^22^

Using new material from the Spanish colonial archives alongside contemporary films, books and press reports, this article shows how the image of Mikomeseng as the embodiment of benevolent colonial rule was constructed by Francoist experts and officials around a brutally repressive institution, one which encapsulated the violence of Spanish colonial rule in West Africa and of the Franco regime as a whole. It will begin by analysing how those behind the establishment of Mikomeseng saw it as a microcosm of the ideal Francoist colonial state, and how it was presented as such in Spanish propaganda and publicity in the decade after 1945. It will then use the protests of 1946 to explore the violence, repression and neglect to which the residents of Mikomeseng were subject, their causes and effects, and the acts of resistance they provoked. Finally, it will show how Mikomeseng, the fight against leprosy and colonial health policy more generally were used to re-establish Spain’s status within the international scientific community following its period of post-war isolation.

## Planning and Propaganda

In 1941, just two years after Franco’s victory in the Spanish Civil War, colonial health authorities in Spanish Guinea began to formulate plans for a new approach to the treatment of leprosy across their territories. In part, their focus on leprosy reflected the relatively high prevalence of the disease. Health officials estimated that there were 3,000–4,000 leprosy sufferers in the region, out of a total population of under 200,000. In comparison, Spain itself contained an estimated 2,700–4,500 leprosy sufferers out of a total population of 25 million.^23^

The focus on leprosy was not, however, purely the result of careful epidemiological analysis. The disease’s religious symbolism meant that it enjoyed a high profile among the profoundly Catholic Francoist administration. Francoist health officials frequently alleged that the disease and its sufferers had been ‘neglected’ or ‘abandoned’ under the Second Republic, insinuating that such neglect was rooted in the Republic’s anti-clericalism.^24^ By focusing on leprosy control, colonial officials and experts could thus exploit the symbolic value of the disease to promote a distinctive Francoist model of colonial rule. In particular, they explicitly identified the new leprosy settlement as an opportunity to construct an idealised microcosm of the Francoist colonial regime.^25^ In a colonial context in which effective government control was in fact relatively limited and genuine social progress non-existent, Mikomeseng would represent what the colonial health service described as a ‘small state within a greater one’, embodying the peace, order and progress which apparently characterised Francoist rule in Africa.^26^

Mikomeseng was intended to encapsulate Francoist imperialism in a number of ways. First, and most importantly, it would establish order and control over the African population. Security was the first priority of the colonial government in Spanish Guinea, which maintained extensive military and paramilitary security forces, administered a complex and authoritarian justice system, and sought to govern the everyday lives of its subjects through the use of internal passports, work permits and restrictive sanitary policies.^27^ Spanish health officials thus approached leprosy first and foremost as a problem of isolation, monitoring and control. Prior to 1941 officials estimated that only a third of the total leper population lived within the leprosy settlements which were scattered across the territory.^28^ Even within these institutions, Spanish officials complained, the absence of rigorous segregation meant that lepers were able to come and go as they pleased, further spreading the disease by making contact and trading freely with the local population.^29^ The lack of available medications, caused both by wartime shortages and the lack of funding, meant that many of those who voluntarily moved to the settlements soon left again.^30^

As a result, plans for improving treatment revolved around securing a more rigorous model of segregation and control. An initial proposal drawn up in 1943 recommended closing all existing treatment centres and converting the small island of Corisco, which lay off the coast of the Rio Muni estuary, into a new leprosy colony.^31^ The idea was eventually rejected as impractical, and instead local authorities decided in 1944 to close the existing inefficient segregation camps around the region and to concentrate all leprosy sufferers in a central settlement at Mikomeseng. The result, according to the colonial health service, would be ‘more rigorous isolation’ of leprosy sufferers, ‘immeasurably improved’ health outcomes, and ‘profound effects on the health work of the country’.^32^

The focus on security and control in Mikomeseng reflected the wider treatment of the African population in Spanish Guinea, where civic and personal freedoms—from property rights to the ability to purchase alcohol—were tightly regulated. But for Spanish officials and experts, Mikomeseng still encapsulated the supposedly progressive nature of Spanish colonial rule. Health authorities were keen to stress both the scientific and the humanitarian credentials of the new facility. Mikomeseng’s new director appointed in 1945, Victor Martínez Domínguez, argued that modern forms of leprosy treatment had evolved beyond traditional models of simple incarceration. New facilities such as Mikomeseng, he claimed, were ‘true sanatoria where the sick find rational care, in many cases achieving considerable relief for their pain and sometimes even an apparent cure’.^33^ A more nuanced understanding of contagion and segregation policies, he argued, had led to ‘a considerable humanization in the life of lepers’, and meant that many now voluntarily presented themselves to the colony rather than having to be forcibly removed from their communities.^34^

Alongside the medical benefits for leprosy sufferers, colonial authorities hoped that the greater level of control they could exert in Mikomeseng would also help to improve moral standards among patients. Without such control, the Director of Health in Spanish Guinea argued, ‘the lack of morals amongst the indigenous peoples leads to frequent sexual contact between the sick and the healthy’, contributing greatly to the spread of the disease.^35^ African sexuality was a recurring concern for Spanish authorities in Guinea, and was often blamed for the territory’s social problems.^36^ Curbing the supposed sexual promiscuity and lack of self-control among the African population was an integral part of what the Spanish saw as their ‘civilizing mission’ in the region, inculcating the moral standards which they regarded as fundamental to a modern developed society. By bringing leprosy sufferers under closer day-to-day control, colonial authorities hoped not only to provide more effective medical care, but to save their subjects from the negative consequences of their own immoral behaviour.

A key part of this humanitarian mission was the aim of preventing the spread of infection from leprosy patients to their new-born children. One of the very first proposals to reform leprosy treatment came from the colony’s Director of Health in 1941, and was justified primarily by the current inability of medical staff to prevent infection to new-born children. ‘We, civilized men with a colonizing duty,’ the Director argued, ‘are allowing human beings to begin life without hope, and before their first smile are condemning them to a world of affliction.’^37^ As a result of these worries, one of the key features of the redesigned settlement at Mikomeseng was a care home just outside the settlement walls where the children of leprosy patients could be removed to prevent infection.^38^ There they would be cared for and educated by missionary nuns, saved from their pathologised African origins and raised as healthy Catholic citizens of the Francoist colonial state. This focus on the health and welfare of children mirrored the emphasis placed on infant and maternal health within Spain itself, where Francoist authorities aimed both to increase the overall size of the population and to ensure that children were raised as healthy, devout and ideologically committed citizens of the ‘New State’.^39^ By preventing the children of leprosy sufferers from contracting the disease, colonial health authorities, like their counterparts in Spain, were thus aiming to forge citizens for the new Francoist state while also demonstrating their credentials as ‘civilised’ colonisers.

The role of missionaries in children’s care reflected the third key aspect of Mikomeseng’s status as a colonial microcosm, namely the centrality of religious orders and the Church within the life of the settlement. Mikomeseng contained a church and a missionary-run school, both of which featured heavily in publicity and propaganda materials showing leprosy sufferers attending mass and reading the bible.^40^ The children’s care home was run by Conceptionist missionaries, underlining the religious symbolism of the settlement’s mission to ‘save’ children from infection and the importance attached to raising faithful Catholic citizens. Mikomeseng’s health facilities were also staffed by both Spanish and African nuns, a largely self-taught group who had received little or no formal medical training.^41^

The prominent involvement of religious orders reflected their dominance of local social services, which had been largely run by Claretian missionaries since the consolidation of Spanish rule.^42^ Both in Spain itself and in the ‘mission countries’ abroad, Spanish religious orders saw leprosy colonies as ideal sites of conversion, and leprosy sufferers as the perfect subjects for their evangelical activities. One lay missionary working in the Spanish leprosy colony of Trillo praised the patients there for their spirit of ‘happiness and resignation’, and claimed they were sacrificing their limbs for their love of God.^43^ The head of the Spanish Church’s Missionary Society, meanwhile, described institutions such as leprosy colonies and orphanages as ideal sites to ‘create the environment, the climate of conversion in the pagan countries’.^44^ Long-term access to patients, particularly children, in an institution where free movement was strictly limited, provided a perfect opportunity for Spanish missionaries to spread the faith, and for Francoist colonial authorities to promote the spiritual dimensions of their ‘civilising mission’.

The organisation of Mikomeseng also mirrored the structure of colonial administration and the colonial economy in Spanish Guinea as a whole, where absolute European rule was cloaked in the language of ‘tribal’ self-government and economic policy was geared towards supporting Spanish autarky. The Governor General of Spanish Guinea nominally ruled the territory through a network of ‘traditional chiefs’, who were legally responsible for enforcing order and security at a local level.^45^ This form of indirect administration allowed Spain to trumpet its commitment to African autonomy and self-government, while freeing it from the burden of providing basic services and masking the fact that the majority of Africans were denied any legal, property or labour rights.^46^ In addition to being self-governing, Spanish Guinea was expected to be largely self-sufficient, requiring minimal financial support from the metropole and supplying raw materials to support the Francoist pursuit of autarky.^47^

Following the same model, Mikomeseng was also supposedly governed by a Council of Chiefs made up of patient representatives responsible for order and administration, and was divided into a system of ‘tribal villages’.^48^ Apart from the Spanish medical staff and missionaries, all other posts were filled by patients, including the nursing team, cooks, internal police force and maintenance staff.^49^ The land cultivated by patients was expected to provide for a majority of the settlement’s food needs. A market allowed residents to trade the food and goods they produced, both amongst themselves and with the outside world, using a special metal currency to limit the spread of infection.^50^ Reflecting Mikomeseng’s alignment with the Francoist quest for autarky, Victor Martínez Domínguez hailed its success at supporting such a large number of leprosy sufferers with such ‘economy for the state’, and suggested it could be used to provide chaulmoogra oil for all of the leprosy settlements on the Spanish mainland.^51^ Far from representing a genuine form of patient-led self-government or self-sufficiency, however, Mikomeseng remained firmly under the control of Spanish authorities. Its gates and boundaries were protected by a unit of the Colonial Guard to prevent escape and maintain isolation, and its statutes made clear that the institution’s Spanish Director enjoyed ultimate and untrammelled authority over all aspects of life inside the settlement.^52^

Mikomeseng’s combination of order, religiously-infused social provision, self-governance and self-sufficiency was designed to perfectly encapsulate Francoist colonial rule in Spanish Guinea. As soon as the settlement had been established it began to feature heavily in Spanish press and propaganda, with a series of films, books and articles produced in the decade after 1945 lauding all of these characteristics. The 1946 film *The Sick of Mikomeseng*, for example, was produced as part of a series of documentaries on Spanish Guinea commissioned by the Spanish Department of Morocco and the Colonies (*Dirección General de Marruecos y Colonias*).^53^ The film, and others like it which focussed on themes of health, presented Spanish Guinea as a ‘pathologised space’ of threatening and disfiguring sickness, but one which the generosity and humanity of the Spanish colonial state and Spanish missionaries were helping to ameliorate.^54^ It touched on many of the themes discussed by those officials involved in planning the settlement, including the role of missionaries and the Church, isolation and security, the tribal organisation and self-sufficiency of the settlement, and the removal of children from their infected parents.^55^ The film was primarily aimed at Spanish audiences, and was screened in Madrid cinemas over the course of the following year.

The narrative around Mikomeseng began to change following the introduction of sulfone treatment at the end of the 1940s. The drugs were first trialled in Spanish Guinea in 1948, and used extensively from 1949. News of their effectiveness, particularly in comparison to previous treatment methods, prompted a wave of voluntary admissions from across the region from leprosy sufferers who had previously resisted attempts to force them into the settlement. Whereas publicity surrounding Mikomeseng had previously been able to point to its humanitarian and civilisational aspirations, but had been unable to provide any concrete evidence of effective medical interventions, the dramatic impact of these new drugs enabled Mikomeseng’s cheerleaders to promote it as both a humanitarian *and* a scientifically advanced endeavour.

This was reflected in a new film, *Sanitary Mission in Guinea*, which was produced in 1953 by the same director who had made *The Sick of Mikomeseng*, this time through the new state documentary film service *No-Do*, and again funded by the Department of Morocco and Colonies.^56^ It appeared alongside a series of books and articles, all written or informed by Mikomeseng’s director, Victor Martínez Domínguez, who was also named as scientific advisor on the film.^57^ All of these works reproduced the themes which had been promoted in the 1946 film, but with a new emphasis on the transformative impact of Spanish medical science on the lives of African leprosy sufferers.

The new film used two techniques in particular to promote Mikomeseng’s humanitarian and scientific credentials. The first was the use of before-and-after images to provide visual evidence of the success of Spain’s civilising mission in Africa. *Sanitary Mission to Guinea* contained lingering images of five Mikomeseng residents, primarily boys and young men, first showing photographs of their faces covered with leprous boils and sores, followed by their healed faces after receiving sulfone treatment.^58^ The images, claimed the narration, provided ‘evidence’ of the treatment, and left ‘no room for doubt’ as to its efficacy.^59^ The same images were reproduced both in press articles and in Victor Martínez Domínguez’s subsequent book.^60^ The impact of Spain’s humanitarian and scientific endeavour was written on the faces and the skin of its African subjects, transformed from the ‘horrendous’ and ‘repugnant’ condition in which they had been found, to healthy, strong and content subjects of Spanish rule.^61^ Their transformation embodied the supposed goals of Spanish colonialism in Africa, namely the development of the native population along the path towards self-determination. As Martínez-Domínguez declared, Spain’s hope was that its subjects would ‘come of age healthy, vigorous and free from scars [*lacras*]’, and that, after an undefined period, ‘another nation born of Spain prays to God in Spanish for the peace of its mother country’.^62^ These young men transformed from disfigured sickness to unblemished good health thus represented the journey which Spanish Guinea would (eventually) make from indigenous backwardness to vigorous, grateful independence.

Alongside these images, *Sanitary Mission to Guinea* and other Mikomeseng propaganda of the early 1950s made heavy use of epidemiological statistics, something which had been absent from *The Sick of Mikomeseng* and other earlier publicity material. Reflecting the involvement of Martínez Domínguez as scientific advisor, over two minutes of the thirteen-minute documentary were dedicated to explaining a graph showing annual leprosy incidence, prevalence and mortality rates as they had developed over the course of the 1940s and 1950s.^63^ The key message delivered by the narration was the change which occurred with the introduction of sulfones in 1948, and the dramatic increase in voluntary admissions it prompted, combined with steadily decreasing mortality rates.

This section of the film did not make for dynamic cinematic viewing; the convoluted explanation as to why the increase in incidence rates was in fact a *positive* reflection of greater reporting rather than an actual increase in the incidence of the disease was a difficult one to communicate. But the importance of the graph, as the narrator explained, was that it ‘expressed such important events scientifically’.^64^ The Spanish mission at Mikomeseng was thus presented as a scientific and scientifically-advanced enterprise, a message reinforced by the prominent attention also given to images of medical instruments and surgical facilities in the institution’s hospital. During an era in which many of Spain’s leading scientists and medical researchers had fled into exile and the Franco regime was frequently denounced for its backwardness and obscurantist traditionalism, Mikomeseng could be used to demonstrate the scientific and technical credentials of Francoist rule.^65^

## The Dark Side of Mikomeseng

The mass protest of leprosy sufferers in 1946, however, revealed a very different side to life in the settlement. The origins of the protest, the reaction from colonial authorities, and the testimonies of those involved suggest that the settlement, and leprosy policy in Spanish Guinea more generally, was characterised as much by violence, neglect and repression as it was by Christian philanthropy and medical care. While Spanish experts trumpeted the lives transformed by the introduction of sulfone treatment in Mikomeseng, they glossed over those lost through brutal transportation policies and the forced removal of infants from their mothers. This hidden history of Mikomeseng thus sheds a very different light on claims that African leprosy treatment demonstrated the benevolence and efficacy of Francoist colonial rule.

The ‘uprising’, as local authorities labelled it, took place on 18 February 1946. The nature and extent of the protests are not entirely clear. Although no deaths or major injuries were reported, local officials claimed that thousands of Mikomeseng residents had taken part in what was an aggressive and violent protest, threatening the life of the Victor Martínez Domínguez and other Spanish staff.^66^ The outbreak of the protests was met with the swift dispatch of a Colonial Guard force to quell disorder and re-establish control over the settlement. According to the local administrator who was sent to Mikomeseng in their wake, the protests had been sparked by anger towards Martínez Domínguez, whom residents claimed was responsible for the regime of forced labour, hunger and physical violence they were subject to. More generally, they complained that many patients had been in Mikomeseng for years, hearing constant promises that they would be cured but without their situation improving in any way. The solution, in their eyes, was the immediate dismissal of Martínez Domínguez and the introduction of a new, more humane regime in the settlement.^67^

Once control of Mikomeseng had been re-established, the Governor General of Spanish Guinea ordered an investigation into the events, during which more detailed allegations emerged. In April, a local Spanish *practicante* (a male medical assistant) working in Mikomeseng wrote to the Governor General warning of the risks of further outbreaks of violence.^68^ In his formal declaration to investigators, he reported that patients in Mikomeseng were being subjected to a systematic campaign of hunger, forced labour and assault, designed in part to prevent them from revealing the truth about conditions there.^69^ The ‘uprising’, he argued, had been nothing more than a reasonable protest against these conditions. In its aftermath he claimed that Martínez Domínguez and the local administrator had redoubled their barbarous treatment of patients, who were regularly subject to beatings and were threatened at gunpoint if they refused to follow orders. As a result, individuals within the camps had been heard discussing plans to attack and kill Martínez Domínguez and other officials.

According to the informant, the Governor General had been systematically misled by conditions on the ground. Despite the widespread publicity about Mikomeseng in Spain and the film which had shown patients being treated in well-maintained concrete buildings, he claimed that medical facilities amounted to a few dilapidated palm huts which were insufficient to care for the number of patients. Most seriously, he alleged that the process of identifying leprosy patients within the population and transferring them to Mikomeseng had been carried out in a brutal fashion, with individuals dying from hunger and mistreatment on the journey, or abandoned to die by the roadside when they could not walk any further.^70^

The response of Martínez Domínguez and of the colonial leadership in Spanish Guinea was twofold. On the one hand, they dismissed the protest from Mikomeseng residents on the grounds of their race and their illness. In his report on the initial protests and his response to the later investigation, Martínez Domínguez denied all the allegations made against him, claiming that patients received sufficient food and basic supplies, and that he had heard no reports of physical punishment or mistreatment.^71^ The protests against him, he argued, were the unfortunate consequence of the fact that on his visits to the centre he was required to reassert the necessary but humane regime of ‘work and discipline’, which in his absence often lapsed into periods of ‘leisure’.^72^ The ‘simplistic logic’ of the indigenous mind had thus led patients to associate him with imposition of work and discipline when they could not grasp the importance of it.

Added to these racial deficiencies, Martínez Domínguez argued, were the psychological effects of leprosy. ‘All of the leper colonies of the world’, he reported, had to deal with the classic irritable character of the leprosy patient, induced both by the knowledge that they suffered from an incurable disease and by the stigma attached to it.^73^ As a result of this double affliction, patients in Mikomeseng had allowed themselves to be led astray by a minority of rebellious troublemakers. The deputy governor responsible for investigating the incident fully accepted these arguments, describing the protests as the result of the ‘scarce mentality’ and lack of ‘powerful reason’ among the African population.^74^

It was these supposed racial and pathological deficiencies which were used to justify the second aspect of the Spanish response to the uprising: repression and punishment. The initial protest had been met with the dispatch of armed security forces stationed nearby. The deputy governor who produced the initial report on the uprising called for the ringleaders to be handed an exemplary punishment in order to avoid similar events occurring in the future, on the grounds that the ‘special psychology of the indigenous’ was unable to ‘comprehend a pardon without concluding that [the offence] was justified’.^75^ In this he was supported by Martínez Domínguez, who also lobbied for an exemplary punishment to avoid undermining the future work and stability of the settlement.^76^

On the basis of this advice, the Governor General decided to dismiss the African nurse who had led the protests, and to commit him to six months in the colony’s disciplinary brigades. He also sentenced the six other patients identified as ‘ringleaders’ to two months in prison and a further six months of forced labour in special disciplinary brigades within Mikomeseng.^77^ Although this was presented as a relatively humane punishment, the severity of the forced labour and the mistreatment of the prisoners at the hands of Mikomeseng officials caused severe hardship for those involved. The convicted six wrote to the Governor General after four months of labour to request a pardon for the remaining period, on the grounds that they were suffering unduly from the punishment as their bodies were ‘full of leprosy and ulcers’.^78^ The request was turned down on the recommendation of Martínez Domínguez, who argued that a pardon would undermine order. The wider claims of mistreatment in Mikomeseng were dismissed, and no action was taken against the institution’s leadership.

These responses reflected the repressive nature of leprosy regulation in Spanish Guinea, and of health services in the colony more widely. Under the guise of the humanitarian imperative to tackle the disease, new leprosy regulations introduced in 1945 had imposed severe punishments on any sufferers who did not voluntarily turn themselves over to colonial authorities, or any relatives, neighbours or tribal ‘chiefs’ who did not report them.^79^ All those suspected of suffering from the disease were required to submit to medical examination every six months, and only those certified as being free from leprosy could access government services or apply for government documents or contracts. Leprosy was thus integrated into the pre-existing regime of sanitary passports which all (African) residents were required to carry, and which contained information about blood type and key diseases such as smallpox and sleeping sickness. These authoritarian sanitary regulations were a central tool in Spanish efforts to exercise control over the territory and over the native population, subjecting local residents to mandatory and intrusive examinations under threat of severe punishments.^80^ The official regulations governing Mikomeseng, which were published at the same time as the new leprosy policies, underlined the dictatorial powers of the health administration there.^81^ All disciplinary measures, the regulations stated, lay solely in the hands of the Director, in this case Martínez Domínguez, who could impose any punishments and sanctions he thought fit. Protest or collective action of any kind was explicitly and strictly prohibited.

The combination of authoritarian control, racist disdain for patients, and a conviction of the humanitarian value of leprosy campaigns underpinned another disturbing underside to life in Mikomeseng: the separation of new born infants from their parents.^82^ As we have seen, the need to protect children from infection had been one of the chief motivations behind the original expansion of the Mikomeseng, and the process of removing new born infants from their mothers and taking them to be raised in the missionary-run children’s home had featured prominently in propaganda about the settlement. Unsurprisingly, however, the policy was fiercely opposed by leprosy sufferers and provoked widespread resistance. In many cases pregnant women attempted to escape the settlement, or to hide evidence of their pregnancy and give birth in secret.^83^

In addition to the distress and medical risks this entailed, the policy also, as Martínez Domínguez himself later admitted, resulted in the avoidable deaths of a number of children. Of the 226 children born in the settlement between 1946 and 1953, 62 were removed from their mothers at birth. Although, as intended, this reduced the rate of infection, 43 of these children died as a result of malnutrition and other diseases exacerbated by the fact that they were not breastfed, a mortality rate of over 69 per cent.^84^ As a result, the policy had to be modified to allow babies to stay with their mothers for the first twelve months, increasing infection rates but drastically cutting infant mortality.

The ability and willingness of Spanish health officials to pursue such harmful policies reflected their particular attitude towards Mikomeseng. For Martínez Domínguez, the settlement was a laboratory, a site of ‘true experiment’ as he called it, in which medical innovations could be tested on a captive and isolated population without fear of professional or political consequences.^85^ This outlook mirrored the wider attitude of the Spanish officials who regarded Mikomeseng as a colonial microcosm, a ‘small state within a larger one’, where they could build a perfect miniature version of Francoist colonial society around a controlled and corralled population, and use it to demonstrate to the world the success of Spain’s ‘civilising mission’ in Africa.

But the reality of life in Mikomeseng was less a reflection of benevolent Spanish colonial rule than it was of the wider brutality of the early Franco regime. This was evident in some of the parallels between Mikomeseng and leprosy settlements on the Spanish mainland. The Valencian settlement of Fontilles was, alongside Trillo, one of the primary Spanish sites for leprosy care and research, and played a prominent role in Francoist publicity and propaganda.^86^ The Franco regime had returned Fontilles to the Jesuits following the Spanish Civil War, and, as in Mikomeseng, religious education and devotion was re-established at the heart of settlement life. But, as in Mikomeseng, the settlement’s authorities struggled to retain control over a distrusted and resentful patient population. The Fontilles leadership suspected that the majority of patients were communist sympathisers, and reported that during the Second World War they avidly followed news of the conflict on the radio, hoping it would lead to a change of regime. Although there were significant differences from the situation in Mikomeseng—the leaders of Fontilles had enjoyed no direct coercive powers and had to petition local civil authorities for help in maintaining order—a strict new regime was established during the height of Francoist repression between 1939 and 1945. Personal possessions and agricultural land were confiscated from suspect patients and redistributed to the ‘deserving’, marriage and mixed-sex interaction between patients was banned, ‘troublemakers’ were expelled and attendance at mass was made compulsory.^87^

Beyond the realm of leprosy control, the coercive apparatus of Mikomeseng also echoed the wider use of health and social policy in Francoist repression. The child separation policy, for example, paralleled similar contemporary practices in mainland Spain, where the children of Republican prisoners were regularly removed from their parents to be brought up in Catholic families or religious institutions in order to save them from political infection, a policy linked to notions of Marxism as both an hereditary affliction and as a ‘contagion’ which needed to be isolated from mainstream society.^88^ This practice formed part of the much wider post-war repression through which the Franco regime sought to purge Spanish society of all traces of the defeated Republican forces and their supporters, and to forge a ‘New State’ around the memory of the victorious Civil War ‘crusade’ and traditionalist Catholic values.

In many ways Mikomeseng thus represented a miniature version of the Francoist state which had been developing on the Iberian Peninsula since 1939. The intolerance of protest and dissent, the violence meted out to those perceived to be challenging the regime, and the neglect of living standards despite the lofty propaganda rhetoric of social justice were all features of the Franco regime which were mirrored within the walls of Mikomeseng. The settlement was indeed a microcosm of Francoist rule, but not in the way its founders intended.

## ‘An example to the world’

Despite the violence of life in Mikomeseng revealed by the 1946 protests, the Franco regime continued to hold it up as an exemplar of its civilized values and commitment to African development. Much of the propaganda built around Mikomeseng and colonial leprosy treatment was aimed at promoting the success of Francoist colonialism to a Spanish audience. But it also increasingly included a comparative international dimension, one which emphasised Spain’s superiority to other European imperial powers while also suggesting that it was Spain’s imperial status which justified its standing within the international community.

In the case of the successful new sulfone treatment, it was clearly impossible to claim that the drugs were the result of a Spanish scientific discovery. Nevertheless, publicity materials suggested that Spain was unique in its willingness to meet the costs of providing new treatment. The *No-Do* film of 1953 highlighted the great costs which the Spanish state had borne in providing medicines and medical supplies to the patients of Mikomeseng.^89^ An article published by the jurist and colonial administrator Rafael Galbe Pueyo in the same year claimed that colonial authorities had paid 4 million pesetas towards the construction of new buildings in the facility, and an additional 2 million pesetas annually to provide sulfones—an act of ‘unprecedented generosity’ from a colonial power.^90^ During the Second World War, officials had worried that the lack of available leprosy medication in Spanish Guinea was prompting many of those who had voluntarily entered leprosy settlements to leave, often crossing over into neighbouring territories to access more effective treatment from American missionaries.^91^ To reinforce the idea of Spanish exceptionalism, Mikomeseng propaganda efforts now emphasised the fact that new patients were arriving from outside of the borders of Spanish Guinea, suggesting that neighbouring territories under British and French control were unwilling or unable to offer similar levels of care.

This comparative argument was made explicit at the end of the *Sanitary Mission to Guinea* film. ‘Do not forget’, began the final scene, ‘that today leprosy is cured! And celebrate as well, the fact that Spain can hold up before the community of nations the work that it is carrying out in the colony of Guinea.’^92^ The international dimension to publicity surrounding Mikomeseng reflected the extent to which the Spanish government and Spanish health officials saw leprosy, and the wider field of colonial health, as a means to demonstrate Spain’s credentials as a civilised and civilising global power. Even before the end of the Second World War, Spain’s leading colonial medical expert, Luis Nájera Angulo, had argued that Spain’s historical affinity and geographical proximity to Africa would make it an indispensable partner in any future efforts to coordinate international and inter-imperial health on the continent.^93^

In the immediate aftermath of the Second World War, the international condemnation of the Franco regime on account of its fascist origins and the diplomatic isolation which followed made the goal of promoting Spain as a civilised colonial power even more urgent. Spain was excluded from the newly-formed UN and its specialised agencies in 1946, as well as from most other international scientific organisations and networks. The technical and humanitarian discourse surrounding international health, however, made it the ideal field for Francoist officials and experts to argue against Spanish exclusion. Global health emergencies such as the post-war polio epidemic provided opportunities for the Franco regime to establish itself within the international system, with Spanish experts helping to legitimise the regime by attending international conferences and working with international organisations.^94^ As the advent of the Cold War began to ameliorate criticism of the Franco regime, the World Health Organization was one of the first international organisations to accept Spanish membership in 1951.^95^

In this context, leprosy’s religious and cultural associations allowed the regime to present itself as both scientifically advanced and socially progressive, while also reminding the international community of Spain’s ‘glorious’ imperial past. As the Director of the Spanish Institute of Colonial Medicine, Valentín Matilla Gomez, argued at the end of 1945, Mikomeseng was an ‘example to the world’, and a key part of the Spanish colonial health system which demonstrated ‘both to neighbouring colonies and to the whole world, that Spain continues to be the generous, selfless, civilizing and evangelizing nation it has always been’.^96^

This international dimension to Spanish leprosy propaganda and the importance of the field in helping to overcome Spain’s post-war isolation was most clearly evident in the International Leprosy Congress held in Madrid in 1953. The congress was one of the first international scientific events ever hosted by Franco’s Spain. Welcoming 400 delegates from over 40 different countries, and supported by the WHO and UNESCO, it was held up by the regime as evidence of Spain’s successful re-integration into the international scientific community. The original decision to host the event in Spain had been taken at the previous conference held in Havana five years earlier, influenced both by the historical ties between the Spanish and Latin American medical communities, and by concerted lobbying by Francoist health authorities.^97^

The organisers of the conference, including leading Spanish leprosy experts, the Spanish Foreign Affairs and Interior ministries, and the Department of Health, also saw it as an opportunity to promote the scientific and social credentials of the regime to the outside world, organising extensive tours of medical facilities and providing evidence to delegates of the supposed triumphs of Francoist public health.^98^ Mikomeseng featured prominently in these efforts, with Martínez Domínguez presenting a paper on the successes of the settlement, and exhibitions, including the ubiquitous before-and-after photographs, promoting Spain’s achievements there.^99^

For Francoist officials such as the Director General of Health, José Palanca, the field of leprosy was an ideal means to promote Francoist medicine to the wider world. Addressing the opening session of the congress, he told delegates that Spain had worked so hard to ensure the international conference came to Madrid because ‘we wanted to show you our medical achievements and our campaign against leprosy, which we in Spain carried out alone, without anyone’s help and despite difficult economic conditions’.^100^ The treatment of leprosy, he argued, was unique in combining ‘a strict scientific character’, with the ‘humanitarian, altruistic and disinterested qualities’ which characterised Spanish medicine.^101^

The uniqueness of Spanish medicine, according to his argument, lay in its combination of advanced technical expertise with the socially progressive and humanitarian principles which underpinned the Francoist state. The Interior Minister, Blas Pérez González, told delegates that the care of lepers was an integral part of Spanish history, highlighting the humanitarian efforts of historical figures from *El Cid* to the *Reyes Católicos*.^102^ This proud history, he argued, was reflected in the expertise of contemporary Spanish leprosy experts and in the advanced state of leprosy treatment under the Franco regime, following its neglect under the Second Republic. The importance of the conference in marking the end of Spain’s post-war isolation was clear from his closing address. By attending the conference, he argued, delegates would be able to learn the ‘truth of Spain’, particularly in the social field, and would help to end Spain’s patient wait for this truth ‘to impose itself on international relations’.^103^ These sentiments were clearly reciprocated by many of the conference’s delegates. Representing the WHO, the Italian tropical medical expert Mario Giaquinto argued that the conference demonstrated ‘the interest that [Spain] has in the solution of health problems, and the value of its cultural and scientific contribution to international efforts’.^104^

The Madrid leprosy conference reflected the wider success of Francoist experts and health authorities in using the field of leprosy to re-establish their status on the international stage. Their ability to mobilise support from Latin American colleagues in order to secure Spain’s hosting of the conference, as well as the large number of Latin American delegates who travelled to Madrid in 1953, demonstrated the important role Latin America played in providing a gateway for Spanish participation in wider forms of international health during the post-war period. A new Ibero-Latin American College of Dematology (*Colegio Ibero-Latinoamericano de Dermatología*; CILAD), for example, which had first been discussed in Havana and was given the green light to expand by delegates at the Madrid conference, was dominated by Spanish leprosy experts and part-funded by the Spanish government.^105^

Beyond the Ibero-American region, leprosy was also the first field in which Francoist experts were able to establish a leading role within international health organisations. The Spanish dermatologist and president of CILAD, José Gay Prieto, was appointed in 1959 as the first head of the WHO Leprosy Unit, leading the organisation’s global campaign against the disease which included extensive work in Africa.^106^ In doing so he was the first Spanish expert, other than exiled Republicans, to be appointed to a senior position within the WHO.^107^ Prieto’s rise reflected the prestigious position Spain enjoyed within the WHO’s anti-leprosy programme more generally. Between 1957 and 1958, for example, recipients of WHO fellowships from as far afield as Turkey, Yugoslavia and French West Africa were sent to Spain to study leprosy treatment, and in 1959 the WHO agreed to implement a leprosy eradication programme across the Iberian Peninsula through its technical assistance programme.^108^

Although Prieto’s professional background was in Spain itself rather than its colonial territories, his international prominence provided opportunities for other Spanish leprosy experts to establish roles within the WHO. Among them was Victor Domínguez Martínez, who despite the brutality of the regime he had presided over in Mikomeseng, went on to work with the WHO Leprosy Unit and sit on the WHO expert committee on leprosy during the 1950s and 1960s.^109^ His professional success reflected the significance of colonial health to the legitimacy of the Franco regime. In the same way that medicine and public health in Spain were used to strengthen the regime’s legitimacy despite the repressive realities of Francoist biopolitics, so Mikomeseng and the treatment of leprosy helped to boost Spain’s international status regardless of the brutality of life both in the settlement itself, and in Spanish Guinea as a whole.

## Funding

This work was supported by the Wellcome Trust-funded project, ‘Reluctant Internationalists: A History of Public Health and International Organisations, Movements and Experts in Twentieth Century Europe’ (PI Jessica Reinisch, grant reference 097779/Z/11/Z) at Birkbeck, University of London.

## Footnotes

1 There is some Spanish-language literature on the history of Mikomeseng, including Francisco Javier Martínez Antonio, ‘Imperio enfermizo. La singular mirada mórbida del primer franquismo en los documentales médicos sobre Marruecos y Guinea’, *Medicina & Historia*, 2009, 4, 1–15, which explores many of the themes covered in this article; Benita Sampedro Vizcaya, ‘La economía política de la sanidad colonial en Guinea Ecuatorial’, *Endoxa: Series Filosóficas*, 2016, 37, 279–98; and Carlos Tabernero Holgado, ‘Cine y procesos de medicalización: documentales médico-coloniales de la posguerra española (1946–1949)', in Serena Brigidi, ed., *Cultura, salud, cine y televisión*. *Recursos audiovisuales en Ciencias de la Salud y Sociales* (Tarragona: URV, 2016), 169–207.

2
*Los enfermos de Mikomeseng*, Hermic Films (1946).

3 Rafael Galbe Pueyo, ‘Mikomeseng’, *Africa*, 1955, 165, 11.

4 Carme Molinero, *La captación de las masas: política social y propaganda en el régimen franquista* (Madrid: Cátedra, 2005); Margarita Vilar Rodríguez and Jerònia Pons-Pons, ‘The Introduction of Sickness Insurance in Spain in the First Decades of the Franco Dictatorship (1939–1962)’, *Social History of Medicine*, 2012, 26, 267–87; Ángel Alcalde, ‘Francoist Veterans and the “New State”: Social Benefits and the Consolidation of the Franco Regime (Spain, 1938–1945)’, in Alessandro Salvador and Anders G. Kjøstvedt, eds, *New Political Ideas in the Aftermath of the Great War* (Basingstoke: Palgrave Macmillan, 2017), 219–39.

5 Frederick Cooper, *Decolonization and African Society: The Labor Question in French and British Africa* (Cambridge: Cambridge University Press, 1996); Joseph Morgan Hodge, *Triumph of the Expert: Agrarian Doctrines of Development and the Legacies of British Colonialism* (Athens, OH: Ohio University Press, 2007); Frederick Cooper, ‘Writing the History of Development’, *Journal of Modern European History*, 2010, 8, 5–23.

6 Florentino Portero, *Franco aislado: la cuestión española, 1945–1950* (Madrid: Aguilar, 1989).

7 Virginia Thompson and Richard Adloff, *The Western Sahara: Background to Conflict* (London: Croom Helm, 1980); Ibrahim K. Sundiata, *Equatorial Guinea: Colonialism, State Terror, and the Search for Stability* (Boulder, CO: Westview Press, 1990); Susana Sueiro Seoane, ‘Spanish Colonialism during Primo de Rivera’s Dictatorship’, in Raanan Rein, ed., *Spain and the Mediterranean since 1898* (London: Frank Cass, 1999), 48–64; María Rosa de Madariaga, *Marruecos, ese gran desconocido: breve historia del protectorado español* (Madrid: Alianza Editorial, 2013).

8 Gonzalo Alvarez Chillida and Eloy Martín Corrales, ‘Haciendo patria en Africa: España en Marruecos y en el Golfo de Guinea’, in Xavier Moreno Juliá and Xosé M. Núñez Seixas, eds, *Ser españoles: imaginarios nacionalistas en el siglo XX* (Barcelona: RBA, 2013), 399–432.

9 Sebastian Balfour, *Deadly Embrace: Morocco and the Road to the Spanish Civil War* (Oxford: Oxford University Press, 2002).

10 Sergio Suárez Blanco, ‘Las colonias españolas en África durante el primer franquismo (1939–1959): algunas reflexiones’, *Espacio, Tiempo y Forma, Serie V, Historia Contemporánea*, 1997, 315–31; Norman J. W. Goda, ‘Franco’s Bid for Empire: Spain, Germany and the Western Mediterranean in World War II’, in Raanan Rein, ed., *Spain and the Mediterranean since 1898* (London: Frank Cass, 1999); Gustau Nerín and Alfred Bosch, *El imperio que nunca existió: la aventura colonial discutida en Hendaya* (Barcelona: Plaza & Janés Editores, 2001).

11 Alicia Campos, ‘The Decolonization of Equatorial Guinea: The Relevance of the International Factor’, *Journal of African History*, 2003, 44, 95–116; Mike Elkin, ‘Franco’s Last Stand: An Analysis of Spanish Foreign Policy Regarding Moroccan Independence in 1956’, *International Journal of Iberian Studies*, 2004, 17, 67–86.

12 Alfonso Botti, *Cielo y dinero: el nacionalcatolicismo en España* (Madrid: Alianza, 1992).

13 Teresa Ballester Artigues and Josep Bernabeu Mestre, ‘Lepra y sociedad en la España de la primera mitad del siglo XX: la Colonia-Sanatorio de Fontilles (1908–1932) y su proceso de intervención por la Segunda República’, *Dynamis*, 1991, 11, 287–344; Rosa Sánchez García, ‘La lucha contra la lepra en la España de primera mitad del siglo XX. Evolución de las estrategias preventivas basada en los avances científicos, sanitarios y sociales’, *Asclepio*, 1994, 46, 79–92; Josep Bernabeu-Mestre and Teresa Ballester-Artigues, ‘Disease as a Metaphorical Resource: The Fontilles Philanthropic Initiative in the Fight Against Leprosy, 1901–1932’, *Social History of Medicine*, 2004, 17, 409–21.

14 John Manton, ‘Global and Local Contexts: The Northern Ogoja Leprosy Scheme, Nigeria, 1945–1960’, *História, Ciências, Saúde-Manguinhos*, 2003, 10, 209–23; Shobana Shankar, ‘Medical Missionaries and Modernizing Emirs in Colonial Hausaland: Leprosy Control and Native Authority in the 1930s’, *The Journal of African History*, 2007, 48, 45–68; Kathleen Vongsathorn, ‘Gnawing Pains, Festering Ulcers, and Nightmare Suffering: Selling Leprosy as a Humanitarian Cause in the British Empire, C. 1890–1960’, *The Journal of Imperial and Commonwealth History*, 2012, 40, 863–78.

15 On the history of modern segregationist approaches to leprosy, see Shubhada S. Pandya, ‘The First International Leprosy Conference, Berlin, 1897: The Politics of Segregation’, *Historia, Ciencias, Saude—Manguinhos*, 2003, 10, 161–77.

16 Megan Vaughan, *Curing Their Ills: Colonial Power and African Illness* (Stanford, CA: Stanford University Press, 1991).

17 Fernando Sergio Dumas dos Santos, Letícia Pumar Alves de Souza and Antonio Carlos Siani, ‘Chaulmoogra Oil as Scientific Knowledge: The Construction of a Treatment for Leprosy’, *História, Ciências, Saúde-Manguinhos*, 2008, 15, 29–46. The emergence of sulfone-resistant leprosy meant that sulfones were later supplanted by multi-drug treatment programmes.

18 On leprosy and the symbolic significance of colonial skin, see John Manton, ‘Leprosy in Eastern Nigeria and the Social History of Colonial Skin’, *Leprosy Review*, 2011, 82, 124–34.

19 Michael Worboys, ‘The Colonial World as Mission and Mandate: Leprosy and Empire, 1900–1940', *Osiris*, 2000, 15, 207–18.

20 Much of this was aimed at the ‘*españolización*’ of the colony. See Gonzalo Álvarez Chillida, ‘Epígono de la Hispanidad. La españolización de la colonia de Guinea durante el primer franquismo’, in Stéphane Michonneau and Xosé M. Núñez Seixas, eds, *Imaginarios y representaciones de España durante el franquismo* (Madrid: Casa de Velázquez, 2014), 103–26.

21 Rosa María Medina Domenech and Jorge Molero Mesa, ‘La ley sanitaria colonial: marco legislativo para el análisis de la medicina colonial española en África’, in Alejandro Diez Torre, ed., *Ciencia y memoria de África: Actas de las III Jornadas sobre ‘Expediciones científicas y africanismo español, 1898–1998’* (Alcalá de Henares: Ateneo de Madrid, 2002), 391–400; Rosa María Medina Domenech, ‘Scientific Technologies of National Identity as Colonial Legacies: Extracting the Spanish Nation from Equatorial Guinea’, *Social Studies of Science*, 2009, 39, 81–112; Vizcaya, ‘La economía política’.

22 Paul Preston, *The Spanish Holocaust: Inquisition and Extermination in Twentieth-Century Spain* (London: HarperPress, 2012). On the repressive use of health and medicine under Francoism, see for example Isabel Jiménez Lucena, ‘El tifus exantemático de la posguerra española (1939–1943): el uso de una enfermedad colectiva el la legitimación del “Nuevo Estado”’, *Dynamis*, 1994, 14, 185–98; Michael Richards, ‘Morality and Biology in the Spanish Civil War: Psychiatrists, Revolution and Women Prisoners in Malaga’, *Contemporary European History*, 2001, 10, 395–421.

23 ‘Un estudio de la Isla de Corisco’, June 1943, (15)18 81/08109, Archivo General de la Administración (AGA), Alcalá de Henares; *Memoria del VI Congreso Internacional de Leprología* (Madrid: Gráficas González, 1954), 51–2.

24
*Director Interino de Sanidad* to *Gobernador General de Guinea*, 6 August 1941, (15)18 81/08109, AGA.

25 The idea of leprosy settlements as colonial microcosm was not unique to Spanish Guinea, and has been explored, for example, in Warwick Anderson’s analysis of US colonial medicine in the Philippines. Warwick Anderson, *Colonial Pathologies: American Tropical Medicine, Race, and Hygiene in the Philippines* (Durham, NC: Duke University Press, 2006), 158–79.

26 ‘Memoria del Servicio Sanitario Colonial, 1944’, (15)18 81/08176, AGA.

27 Alicia Campos Serrano, ‘El régimen colonial franquista en el Golfo de Guinea’, *Revista Jurídica Universidad Autónoma de Madrid*, 2000, 3, 79–108.

28
*Director Interino de Sanidad* to *Gobernador General de Guinea*, 6 August 1941, (15)18 81/08109, AGA.

29
*Director de Sanidad* to *Gobernador General de Guinea*, 1 January 1943, (15)18 81/08109, AGA.

30 Untitled report, Víctor Martínez Domínguez, 20 December 1942, (15)18 81/08109, AGA.

31
*Director de Sanidad* to *Gobernador General de Guinea*, 1 January 1943; ‘Un estudio de la Isla de Corisco’, June 1943, (15)18 81/08109, AGA.

32 Memoria del Servicio Sanitario Colonial, 1944’, (15)18 81/08176, AGA.

33 Untitled report, Víctor Martínez Domínguez, 20 December 1942, (15)18 81/08109, AGA.

34
*Ibid*.

35
*Director de Sanidad* to *Gobernador General de Guinea*, 1 January 1943, (15)18 81/08109, AGA.

36 Gustau Nerín, *Guinea Ecuatorial, historia en blanco y negro: hombres blancos y mujeres negras en Guinea Ecuatorial, 1843–1968* (Barcelona: Ediciones Península, 1998).

37
*Director Interino de Sanidad* to *Gobernador General de Guinea*, 6 August 1941, (15)18 81/08109, AGA.

38 Separation of children from their leprous parents was a relatively widespread global practice at the time, carried out to a greater or lesser extent in the Philippines, Latin America and mainland Spain. See, for example, Warwick Anderson, *Colonial Pathologies*, 159; Vicent Comes Iglesia, ed., *Cuidados y consuelos. Cien años de Fontilles (1909–2009)* (Valencia: Biblioteca Valenciana, 2009), 304–8.

39 Josep Bernabeu-Mestre and Enrique Perdiguero-Gil, ‘At the Service of Spain and Spanish Children: Mother-and-Child Healthcare in Spain During the First Two Decades of Franco’s Regime (1939–1963)’, in Iris Lowry and John Krige, eds, *Images of Disease: Science, Public Policy and Health in Post-War Europe* (Luxembourg: Office for Official Publications of the European Union, 2001), 167–86.

40 Martínez Antonio, ‘Imperio enfermizo’.

41 Víctor Martínez Domínguez, *Estudio epidemiológico y clínico de la endemia de lepra en la Guinea española* (Madrid: Instituto de Estudios Africanos, 1954), 22. Efforts to professionalise missionary medicine did not begin in Spain until the late 1940s, and even then had a relatively limited reach.

42 Gonzalo Álvarez Chillida, ‘Les missions clarétaines et l’administration coloniale en Guinée espagnole’, *Histoire, Monde et Cultures religieuses*, 2015, 31, 113–31.

43 ‘Prácticas de enfermeras misioneras seglares en la leprosería de Trillo’, *Salus Infirmorum*, June 1951, 17, 22.

44 Ángel Sagarmínaga, ‘¿Podemos ser misioneras … ?’, *Salus Infirmorum*, 1948, 8, 2–3.

45 Campos Serrano, ‘El régimen colonial franquista’.

46 Sundiata, *Equatorial Guinea*, 32–3.

47 Botti, ‘Cielo y dinero’; Suárez Blanco, ‘Las colonias españolas en África’.

48 ‘Memoria del Servicio Sanitario Colonial, 1944’, (15)18 81/08176, AGA. The policy of dividing leprosy settlements along ‘tribal’ lines was shared across colonial Africa. See Vaughan, *Curing Their Ills*.

49 Valentín Matilla Gómez, ‘Una expedicion científica a la Guinea’, *Medicina Colonial*, November 1945, 4, 330.

50 Rafael Galbe Pueyo, ‘Mikomeseng’, *Africa*, 1955, 165, 9–11. This special currency featured heavily in publicity material about Mikomeseng.

51 Untitled report, Víctor Martínez Domínguez, 20 December 1942, (15)18 81/08109, AGA.

52 ‘Memoria del Servicio Sanitario Colonial, 1944’, (15)18 81/08176, AGA; Martínez Domínguez, *Estudio epidemiológico*, sec. appendix.

53
*Los Enfermos de Mikomeseng*, Hermic Films (1946), Filmoteca Española. On the film crew’s expedition to Spanish Guinea, see Pere Ortin and Vic Pereiró, *Mbini: Cazadores de imagenes en la guinea colonial* (Barcelona: Altair, 2006).

54 Martínez Antonio, ‘Imperio enfermizo’, 10.

55 For an insightful discussion of the film and its significance within early Francoism, see Martínez Antonio, ‘Imperio enfermizo’.

56
*Misión sanitaria en Guinea*, *No-Do* (1953), Filmoteca Española. On the history of *No-Do*, see Araceli Rodríguez Mateos, *Un franquismo de cine: la imagen política del régimen en el noticiario NO-DO (1943–1959)* (Madrid: Rialp, 2008).

57 Rafael Galbe Pueyo, ‘Mikomeseng’, *Africa*, 1955, 165, 9–11; Martínez Domínguez, *Estudio epidemiológico*.

58
*Misión sanitaria en Guinea*, 7.21–8.23.

59
*Misión sanitaria en Guinea*, 8.19.

60 Rafael Galbe Pueyo, ‘Mikomeseng’, *Africa*, 1955, 165, 9.

61
*Ibid*.

62 Martínez Domínguez, *Estudio epidemiológico*, 113.

63
*Misión Sanitaria en Guinea*, 4.45–7.15.

64
*Ibid*., 4.51.

65 Josep Lluís Barona, ed., *El exilio científico republicano* (València: Publicacions de la Universitat de València, 2010).

66 Untitled report, *Administrador territorial de Mikomeseng*, 6 March 1946, ‘Incidente en la Leprosería de Mikomeseng’, 15(18) 81/08109, AGA.

67
*Ibid*.

68 José Luis Martínez Díaz to *Gobernador General de Guinea*, 10 April 1946, ‘Incidente en la Leprosería de Mikomeseng’, 15(18) 81/08109, AGA.

69 ‘Declaracíon de José Luis Martínez Díaz, Bata’, 3 June 1946, ‘Incidente en la Leprosería de Mikomeseng’, 15(18) 81/08109, AGA.

70
*Ibid*.

71 ‘Declaracíon de Dr. Martínez Domínguez’, undated, ‘Incidente en la Leprosería de Mikomeseng’, 15(18) 81/08109, AGA.

72 Untitled report, Víctor Martínez Domínguez, 4 March 1946, ‘Incidente en la Leprosería de Mikomeseng’, 15(18) 81/08109, AGA.

73
*Ibid*.

74
*Subgobernador de la Guinea Continental Española* to *Gobernador General de Guinea*, 4 March 1946, ‘Incidente en la Leprosería de Mikomeseng’, 15(18) 81/08109, AGA.

75
*Ibid*.

76 Untitled report, Víctor Martínez Domínguez, 4 March 1946, ‘Incidente en la Leprosería de Mikomeseng’, 15(18) 81/08109, AGA.

77
*Gobernador General de Guinea* to Víctor Martínez Domínguez, 30 March 1946, ‘Incidente en la Leprosería de Mikomeseng’, 15(18) 81/08109, AGA.

78 Note contained in correspondence between *Subgobernador de la Guinea Continental Española* and *Gobernador General de Guinea*, 10 July 1946; ‘Incidente en la leprosería de Mikomeseng’, 15(18) 81/08109, AGA.

79 ‘Reglamento vigente de la lucha contra la lepra’, *Boletín Oficial de los Territorios del Golfo de Guinea* (15 April 1945).

80 Medina Domenech, ‘Scientific Technologies’; Vizcaya, ‘La economía política’.

81 ‘Reglamento del régimen interno de la leprosería central’, *Boletín Oficial de los Territorios del Golfo de Guinea* (15 April 1945).

82 Martínez Antonio, ‘Imperio enfermizo’; Vizcaya, ‘La economía política’.

83 Martínez Domínguez, *Estudio epidemiológico*, 42.

84 Martínez Domínguez, *Estudio epidemiológico*, 42–4.

85 Martínez Domínguez, *Estudio epidemiológico*, 44.

86 Comes Iglesia, *Cuidados y consuelos*, 272–316.

87 Comes Iglesia, *Cuidados y consuelos*, 293–6.

88 Ricard Vinyes Ribas, Montserat Armengou Martín and Ricard Belis i Garcia, *Los niños perdidos del franquismo* (Barcelona: Plaza & Janes, 2002); Michael Richards, ‘Spanish Psychiatry c. 1900–1945: Constitutional Theory, Eugenics, and the Nation’, *Bulletin of Spanish Studies*, 2010, 81, 823–48. The parallels between the treatment of African leprosy sufferers and Spanish Republicans, particularly in relation to children, are made in Martínez Antonio, ‘Imperio enfermizo’, 14–15.

89
*Misión sanitaria en Guinea*, 10.45.

90 Galbe Pueyo, ‘Mikomeseng’, 11.

91 Untitled report, Víctor Martínez Domínguez, 20 December 1942, (15)18 81/08109, AGA.

92
*Misión sanitaria en Guinea*, 12.57.

93 Luis Nájera Angulo*, ‘*La sanidad pública y la colonización africana’, *Revista de Sanidad e higiene publica*, July 1944, 18, 298–304.

94 María Isabel Porras, María José Báguena and Rosa Ballester, ‘Spain and the International Scientific Conferences on Polio, 1940s-1960s’, *Dynamis*, 2010, 30, 91–118; María Isabel Porras and others, eds, *El drama de la polio: un problema social y familiar en la España franquista* (Madrid: Catarata, 2013).

95 Formal membership of the WHO was granted in May 1951, the second of the UN’s specialised agencies to grant admission to Franco’s Spain following the Food and Agriculture Organization in April 1951.

96 Valentín Matilla Gómez, ‘Una expedicion científica a la Guinea’, *Medicina Colonial*, November 1945, 4, 330–5.

97 ‘Sanitaria internacional’, *Ser*, 1948, 71, 27–8.

98 ‘Memorias generales de la Dirección de Relaciones Culturales, 1954–1957’, (15)18 81/14261, AGA; *Memoria del VI Congreso Internacional de Leprología*, 50.

99
*Memoria del VI Congreso*, 27–9, 1104–204.

100
*Memoria del VI Congreso*, 51.

101
*Memoria del VI Congreso*, 50.

102
*Memoria del VI Congreso*, 126–9.

103
*Memoria del VI Congreso*, 128–9.

104
*Memoria del VI Congreso*, 47.

105 F. M. Camacho, ‘Historia del CILAD. Evolución y perspectivas de la formación de los médicos residentes’, *Medicina Cutánea Ibero-Latino-Americana*, 2008, 36, 312–19.

106 Eduardo Fonseca-Capdevila, ‘Apuntes para una biografía del Dr. José Gay Prieto (II). La dermatología y venereología de Gay’, *Piel*, 2006, 21, 54–60.

107 The former Republican politician, ambassador and Director General of Health, Marcelino Pascua, was head of the WHO’s statistics department from 1948.

108 Project Files, Spain 27, Leprosy, World Health Organization Archives, Geneva.

109 José Gay Prieto and Víctor Martínez Domínguez, ‘International Work in Leprosy, 1948–1959’, *WHO Chronicle*, 1960, 14, 3–39; Dirección General de Plazas y Provincias Africanas, *Labor sanitaria en las provincias de la región ecuatorial* (Madrid: Fénix-Artes, 1961), 1.

